# Bioaerosol assessment in indoor and outdoor environments: a case study from India

**DOI:** 10.1038/s41598-023-44315-z

**Published:** 2023-10-23

**Authors:** Raisa Jabeen, Mohamed Ibrahim Kizhisseri, S. N. Mayanaik, Mohamed Mostafa Mohamed

**Affiliations:** 1 Department of Environmental Engineering, China State Construction Engineering Corporation, Middle East L.L.C, Dubai, United Arab Emirates; 2 Department of Civil and Environmental Engineering, United Arab Emirates University, Al Ain, Abu Dhabi, United Arab Emirates; 3 B M S College of Engineering, Bengaluru, India

**Keywords:** Environmental impact, Environmental impact

## Abstract

Exposure to bioaerosols has been associated with the occurrence of a variety of health impacts, including infectious illnesses, acute toxic effects, allergies, and cancer. This study aimed at evaluating airborne bacteria and fungi populations at different indoor and outdoor sites on a college campus in Bengaluru, India. Bioaerosol samples were collected using a two-stage Andersen air sampler; and isolates were identified using standard procedures. Six air samples and meteorological data were collected in March and April 2014 to examine the effects of temperature and relative humidity on bioaerosol concentration using linear regression modeling. Among all sites, the canteen showed the highest bioaerosol levels both indoors and outdoors. Specific bacterial identification was not possible, but gram staining and microscopic analysis helped to identify gram positive and gram negative bacteria. The most prevalent fungal species in the samples were *Cladosporium*, *Aspergillus niger*, *Penicillium*, *Rhizopus*, *Fusarium*, *Mucor*, and *Alternaria*. Due to the impact of weather conditions, such as temperature and relative humidity, the bioaerosol concentration varied greatly at each site according to the regression model. The indoor bioaerosol concentrations at all sites exceeded the values established by the American Industrial Hygiene Association (< 250 CFU/m^3^ for total fungi and < 500 CFU/m^3^ for total bacteria). Higher concentrations of bioaerosols may be attributed to the transportation of microbes from the ground surface to suspended particles, the release of microbes from the respiratory tract, higher rate of shredding of human skin cells, and many other factors.

## Introduction

Although rapid urbanization and industrialization have contributed to economic expansion, air quality has significantly declined due to increased air contaminants^1^. The atmosphere is a dynamic system that consists of particulate matter and microorganisms. Microorganisms are ubiquitous and significantly affect the ecosphere. Most microbes originate from natural sources, including sand, ponds, animals, and human beings^2^. In addition, they are released into the atmosphere through agricultural operations, healthcare institutions, hospitals, and industrial processes, such as wastewater treatment, animal rendering, food processing, and fermentation^3^. The microorganisms dispersed in the atmosphere are sensitive indicators of environmental quality. Physical and chemical factors, such as heat, viscosity, light, organic and inorganic particle suspensions, food availability, and human activities, affect the variety of species and the number of organisms existing in the atmosphere. Humans inhale microorganisms in the form of bioaerosols from the air^4^. Generally, bioaerosols are colloidal suspensions comprising airborne liquid droplets and solid particles^5^ derived from plants (pollens) and animals. They might contain pathogenic and/or nonpathogenic dead or live microorganisms (bacteria, viruses, and fungi)^6^. Atmospheric aerosols are mainly composed of cells; cell fractions; or organic matter of animal, plant, and microbial origin (approximately 50% of all aerosol particles)^7^. As airborne particles are lightweight, they can be readily moved, transferred, and shifted from one environment to the other^8^. The size of bioaerosols ranges from approximately 0.05–100 µm based on their origin, aerosolization technique, and environmental factors at the site. The inhalable fraction (PM 2.5) is the most significant component as it easily penetrates the alveoli of the lungs. The particles accumulated in the lungs can be easily transferred to the blood and then quickly spread throughout the human body, including babies^9^. Thus, exposure to bioaerosols from indoor and outdoor environments is of significant concern^10^.

Sources of indoor airborne biological pollutants include outdoor air, human bodies, wallpaper, carpet, suspended particles, air conditioners, and animal excreta^11^. Specific human activities, such as talking, sneezing, coughing, walking, washing, and toilet flushing, further increase the airborne microbial load. The growth and multiplication of bioaerosols in the indoor atmosphere are enhanced by environmental factors, such as building structures and location, temperature, humidity^12,13^, air exchange rate^14^, air movement, incompetent design, air circulation system, and interior space^15^. In indoor buildings, key sources of microbial proliferation and dispersion include air conditioners, fans, coolers, and humidifiers. On average, people breathe in 10 m^3^ of air every day. Moreover, they are constantly exposed to airborne microbes as they mostly spend their time (90% or above) indoors, such as in homes, schools, university dorms, stores, cars, airplanes, and workplaces^16^. Increasing evidence suggests that exposure to these conditions with insufficient ventilation and degraded air quality may result in the development of infectious diseases, allergic and irritant reactions, respiratory disorders, hypersensitivity responses, or even the “sick building syndrome,” correlated with occupational stress and characterized by symptoms of unclear etiology, including eye, nose, throat, and skin irritations; headache; and fatigue^11,17^.

As the external and internal environments are interconnected, microorganisms might spread randomly^6^. Notably, the location and weather conditions such as rain, snow, or dust storms majorly influence the outdoor microbial concentrations because the transmission of bioaerosols in these environments is primarily driven by hydrodynamic and kinetic factors^18^. The bioaerosols can be dispersed from the source to the receptor site under the influence of wind, and scavenged from the air either by precipitation or by direction deposition to the surface. Also, some human activities can let to the spread of bioaerosols in outdoors. During the atmospheric transport process, bioaerosols may interact with atmospheric ions, solar radiation, and external factors which can further lead to the physical and chemical transformation of bioaerosols^19^. They can also alter the climate, visibility, and quality of life^1^. Bacteria are broadly classified as gram-positive and gram-negative. Gram-positive bacteria are predominant in indoor spaces, and their presence indicates poor air quality. They are shed into the surroundings through human skin and respiratory secretions. At high levels, gram-negative bacteria increase the risk of contracting respiratory diseases due to the presence of endotoxins in their outer membrane, which affects lung function^20^. Fungi are ubiquitous in indoor and outdoor environments. However, they do not proliferate in the air but rather grow well on surfaces or substrates. Building constituents, such as ceiling tiles, wall covering, wood, and sand bricks, provide suitable substrates for fungal proliferation, especially when moist. *Cladosporium*, *Alternaria*, *Penicillium*, and *Aspergillus* are the most common fungi that cause respiratory tract allergies^21^. Therefore, the presence of microbial fractions (bacteria and fungi) in both outdoor and indoor habitats is a significant threat to the environment and human health^22^.

In many developed nations, the scientific database on exposure to bioaerosols at home and work has grown lately to assess how this exposure affects health^10^. Although few reports have shown the effects of exposure to bioaerosols in residential^22^ and nonresidential settings, such as food courts^23^, the data regarding bioaerosol exposure at educational institutions in India is lacking. Over six million students graduate from Indian universities each year. According to previous studies, the indoor atmosphere significantly affects the students’ well-being and academic grades^24,25^. Due to higher population density and crowded campuses, the likelihood of sick students being exposed to and spreading biological contaminants may be higher in colleges in India than in those in any other developed nation. Therefore, it is crucial to evaluate the indoor and outdoor biological pollutants on university campuses in India. Many studies have been conducted to determine the concentrations of airborne bacteria at various indoor sites in academic institutions, including libraries^26,27^, classrooms^28–31^, dormitory rooms^15^, archives^32^, and labs^33,34^. However, these studies mainly focused on a specific environment.

Studies on the microbiological analysis of indoor and outdoor air quality are limited^20,33^. In the present study, we evaluated different species of airborne bacteria and fungi and their corresponding levels along with the environmental parameters in various indoor and outdoor environments (classroom, washroom, canteen, library, environmental laboratory, and seminar hall) where students and professors spend most of their daily time. The microbiological content in indoor and outdoor air effects have an influence on the physical and mental health of these individuals, as well as on the hydrological cycle, temperature, and visualness.

## Materials and methods

### Study area

BMS College of Engineering is located in Bengaluru, the capital city of Karnataka, India. Bengaluru has a tropical savanna climate and due to its high elevation, it enjoys a more moderate climate throughout the year. Bengaluru receives rainfall from both the northeast and the southwest monsoons. The city experiences the winter season from December to February with a chilling breeze, and summer season from March to May and the misty monsoon season from June to September. The university is situated at 12° 56′ 31.7″ N and 77° 33′ 57″ E and has an area of 11 acres and 3035 m^2^, with a built-up area of 99,500 m^2^. The student population at this institution is the highest among all engineering colleges in Karnataka. Currently, approximately 5000 students are pursuing higher education at this institution. It consists of a trust office, administrative block, B S Narayan Platinum Jubilee block, P.G block, data Centre, BMS College of architecture, BMS College of Law, Mechanical Block, Sports complex, Canteen, ladies hostel, science block, boy’s hostels and a bank.

### Sampling locations

**Figure 1:**
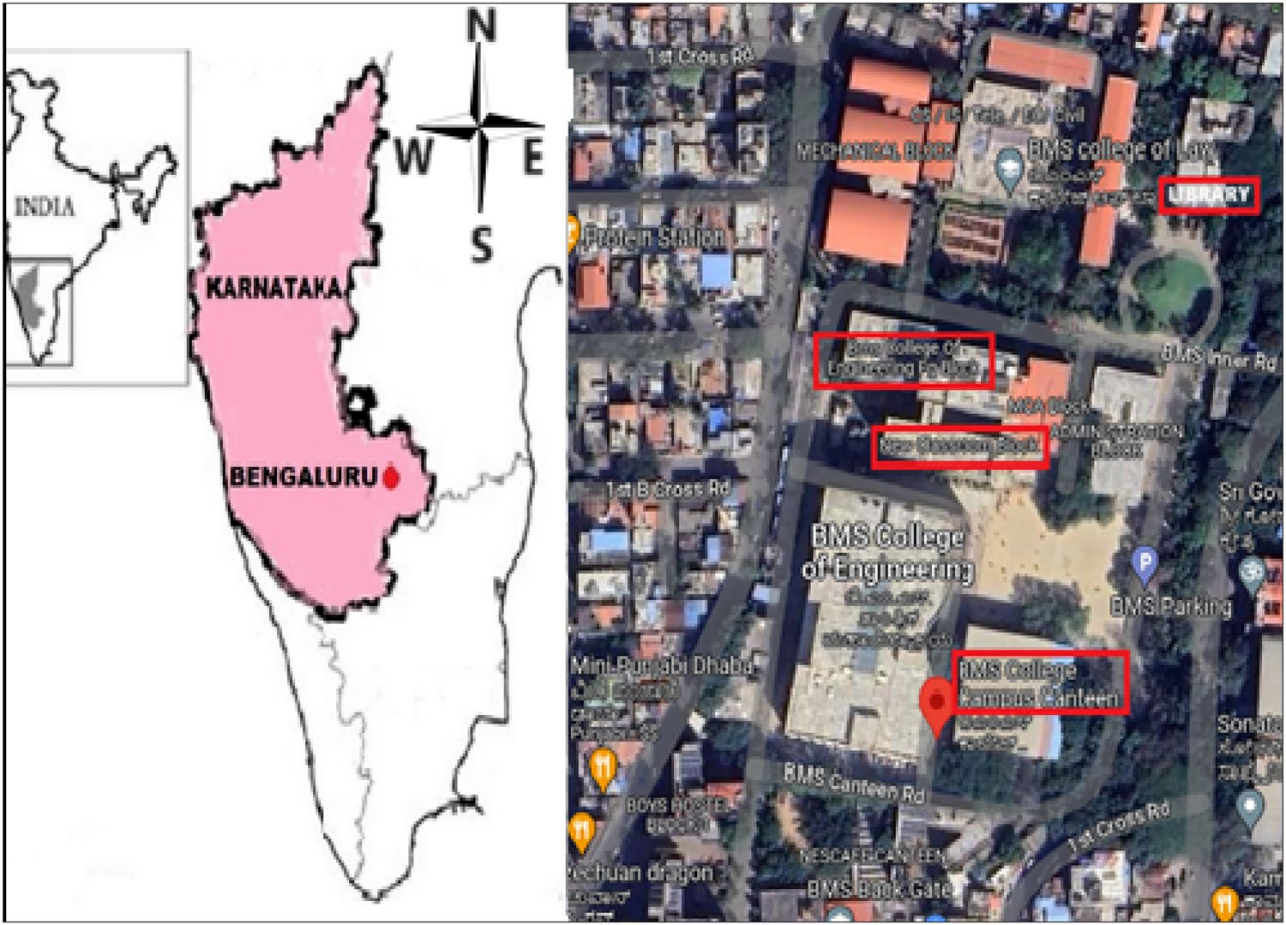
Sampling sites at the university campus study.

Sampling was conducted in six locations: Classroom, washroom, canteen, laboratory, library, and seminar hall. Due to the challenges to sample from every location, the samples were collected from the entrance (library), center (classroom, washroom, laboratory, and seminar hall), and at the rear (canteen) of the college campus with different occupancy levels. These sites were expected to indicate total exposure to bioaerosols at the university. The description and the characteristics of the sampling sites are depicted in Fig. 1 and Table 1. During the sampling process, all windows at the locations were found to be closed, and the doors of the classroom and seminar hall were closed as well. However, the doors at the canteen and washroom were open, and the door at the library was partially opened for easy access to students.

**Table 1:** Description of the sampling sites.

Sampling characteristics	Classroom	Washroom	Canteen	Library	Environmental lab	Seminar hall
Sampling site location	First floor	First floor	Ground floor	First floor	First floor	First floor
Size (m^2^)	50–100	15–20	150–200	2625.67	30–40	60–70
Occupancy of students during sampling	Zero	10–15	25–50	25–30	3–6	Zero
Ventilation	Natural and mechanical	Natural and mechanical	Natural and mechanical	Mechanical	Natural and mechanical	Mechanical
Number of windows	6	4	20	15	8	3
Outdoor door	1	1	2 (front and back)	1	1	1
Hygiene	Good	Good	Average	Good	Good	Average

### Sampling procedure

**Figure 2:**
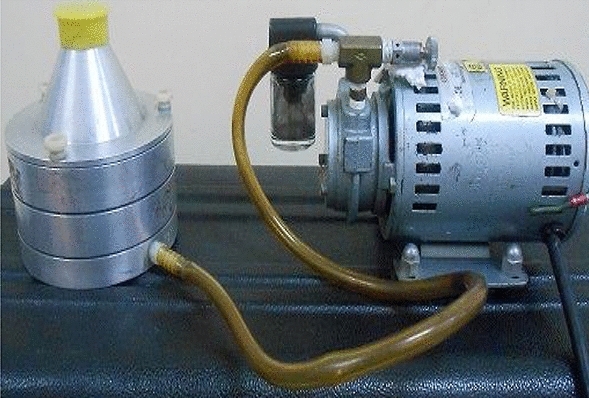
Two-stage Andersen sampler.

A two-stage Andersen sampler as shown in Fig. 2, consisting of two metallic plates of 200 holes per plate with pore size of upper larger 8.0 µm and smaller size of 0.95 µm respectively, was employed to collect bioaerosol samples. The samples were collected by sucking the air at the rate of 28.3 l/min for 2 min from March to April 2014 with three repetitions each time. These months were chosen as they mark the transition from winter to summer with rising temperatures. A total of 72 samples were collected including 36 indoor samples and 36 outdoor samples of bacteria and fungi. The sampling time was between 1.30 pm and 2.30 pm, as this period was ideal for making comparisons of indoor bioaerosols levels at different places with various occupancy rates. All indoor and outdoor air samples were collected from the normal breathing level at a height of 4–5 feet above the ground by placing the sampler at the center of the room for indoor samples and at least 2 m away from the door entrance of the sampling sites for outdoor sampling.

Nutrient agar was used for culturing bacteria, whereas Rose Bengal agar was used for culturing fungi. The culture medium was prepared in the laboratory by weighing 3.9 g of nutrient medium per 100 ml of distilled water and the mixture was homogenized in the media bottle. The culture medium and the Petri plates were sterilized in an autoclave. The workspace of the laminar flow chamber and the hands were cleaned with ethyl alcohol and the sterilized media was poured into the sterilized Petri plates in front of the flame to prevent contamination and left to solidify at room temperature in a laminar airflow. Before each sampling, the sampler and the hands were disinfected and dried using 70% ethyl alcohol to remove any initial contamination. At each stage of the sampling apparatus, a collecting plate was inserted without its lid. The air to be sampled first passed through the jet orifices in Stage I and then moved through those in Stage II at a faster orifice velocity than that at Stage I. Hence, there was a higher inertial impact on the smaller particles on the agar plate in Stage II than in Stage I. This retained the viable particles on the agar plates and enabled the transport of exhaust air to the vacuum source through the instrument’s bottom output and vacuum hose. The agar collecting plates were removed from the sampling equipment after the sampling was completed. The Petri dishes were covered, and the sampling duration along with the sample and stage number was noted. To avoid condensation drip, the plates were inverted, and their edges were sealed with paper tape. After wrapping the plates in foil, they were transported to the laboratory in an air-tight box. To avoid accidental contamination during experimenting and to check the sterility of the culture media, laboratory and field blanks were prepared. Three laboratory and two field blanks for each set of samples were provided and were used as a negative control to correct the results. For fungal and bacterial samples, the plates were incubated at room temperature and 37 °C, respectively, for 72–120 h in an inverted position. The number of colonies on each plate was counted after incubation using a standard bacterial colony counter. Estimated concentrations of bacteria and fungi in the air were measured and represented as colony-forming units per cubic meter of air (CFU/m^3^).

### Meteorological parameters

Meteorological parameters, such as temperature (°C) and relative humidity (RH%), for indoor and outdoor environments, were recorded using portable equipment such as multi-purpose Hygro-Thermometer model: Extech RH300 and are shown in Table 2.

**Table 2:** Average meteorological parameters recorded on sampling days.

Sampling location	Indoor parameters		Outdoor parameters	
	Temp. (°C)	RH (%)	Temp. (°C)	RH (%)
Classroom	25.9	35.6	23.85	28
Washroom	29.8	44.2	28.1	32.5
Canteen	31.9	54.3	26.95	33.5
Library	28.8	42.8	29.35	41.5
Environmental lab	29.5	49.7	27	57
Seminar hall	31.1	41.3	28.65	54.5

### Microbial identification

The microscope used for bacterial and fungal genes identification in this study is Olympus CH20 biological medical microscope. Bacteria were identified based on macro and microscopic examinations. Crystal violet was used as the primary color for the gram staining test, which was used to differentiate the bacterial isolates into gram-positive and gram-negative categories. A thin film of bacterial culture was placed on a clean slide and stained for 1 min with an ammonium oxalate crystal violet solution for bacterial identification. The slide was then rinsed with water for less than 2 s to remove excess discoloration before being submerged in an iodine solution for 1 min, washed with water, and dried. It was then soaked in 95% ethanol for 30 s and then in a safranin solution for 10 s. Glycerin was added after a final water wash, and it was covered with a cover slip before microscopic testing and optical identification. Bacteria that retained the primary color and appeared purple-brown under a microscope were classified as gram-positive, whereas those that did not retain the primary stain and appeared red under a microscope were identified as gram-negative. By microscopic analysis, all bacterial taxa were recognized and categorized based on colony morphology and cellular form. This approach could not be used to identify particular species.

Fungal colonies were identified based on their morphological characters. However, further identification of the genus level was done by microscopic examination. A small portion of a fungal colony was collected and deposited on a slide containing 4% NaCl using an inoculum loop. A drop of cotton blue stain was instantly applied over it and left for around 1–2 min. After that, the region was covered with a cover slip and prepared for microscopic analysis and visual identification.

### Modeling approach

For statistical analysis, Regression and Pearson Correlation were determined with Microsoft Excel 2016 and IBM SPSS Statistics 29.0.1.0 software.

## Results and discussion

### Bacterial bioaerosol concentration in indoor and outdoor air

The quantity of microorganisms detected in the college's air samples varied with the sampling sites. Table 3 displays the computed indoor and outdoor findings for contamination of bacteria, along with average number of occupants at each sample location. The average indoor total bacteria count (TBC) ranged between 924 and 2750 CFU/m^3^ at various sampling points. Among all the sampled locations, the canteen had the highest average indoor bacteria count of 2750 CFU/m^3^, followed by washroom (2647 CFU/m^3^), environmental laboratory (1998 CFU/m^3^), classroom (1709 CFU/m^3^), seminar hall (1695 CFU/m^3^) and the least average indoor bacteria counts were noticed in the library with levels of 924 CFU/m^3^. Our findings were in line with a research by^35^ that examined the microbiological air pollution in libraries, cafeterias, and a selected classrooms at the two schools. They found that cafeterias had the greatest amounts of bacteria and libraries had the lowest levels. The canteen is a hotspot meeting area for the students and lecturers during the leisure, a place to have a meal and to relax. Since the monitoring was done during peak hours, variables such as high occupant density, poor ventilation, and unsanitary conditions were observed, which may be the cause of the canteen's higher indoor bacterial counts. Studies performed by^36^ found that among five sampled locations in the university campus, café and cafeteria had the highest average bacterial concentrations. Furthermore, studies by^37^ on bioaerosols in different indoor sites of college showed a similar result to our study. Toilet flushing is one of the major cause of bioaerosol emission in the toilets by aerosolizing feces from the movement of toilet water during a flushing event (i.e., bubbling, swirling, splashing)^38^.The Chan et al.^39^ studied the bioaerosol from the washrooms of the office building and reported that the indoor airborne bacteria increased from morning to evening due to the high occupancy load. This results can be correlated with our studies as the occupant density was highest at the time of sampling. Occupant density is one of the major factors that contribute to the indoor bioaerosols and as seen from the Table 3, an increase in bioaerosol concentration was noticed with the increase of occupants, except for the library. This may be as a result of the library's larger surface area and the fact that the sampling was done at its center. However, sampling at different sections of the library could give the more information on the bioaerosols emissions. Human traffic, human activities such as conversation, coughing and sneezing and air currents by the fans cause dust particulates to suspend and eject bioaerosols to the indoor air^40^. Srivastava et al.^41^ studied size segregated bioaerosols at different sites of JN university and found that the environmental laboratory had an average indoor bacterial count of 222.6 CFU/m^3^, which was significantly lower than the findings of the current study of average indoor bacterial counts in environmental laboratory. The environmental lab's doors were open while samples were being taken, which could be one of the reasons for the higher concentrations in this area since bioaerosols can enter the labs from the outside environments. Other potential causes include the cleaning frequency, which was done twice a week, and the unintentional release of aerosols during experimentation. Several research has been done related to indoor bacterial counts in the classrooms. A few studies are^42–44^ and these researches reported the bacterial densities between 359.6 and 2588 CFU/m^3^ when the classrooms were occupied. Despite the fact that the classrooms were empty, the average indoor bacteria count for the current study was within this range. This may be because of less air movement between the indoor and outdoor environments, as the windows and doors were found shut throughout the sampling days. Additionally, it's likely that particular human actions at peak occupancy rates caused the bacterial aerosol to be concentrated in this area. Dang et al.^43^ studied the indoor bacterial densities in lecture halls with approximately 300 student occupancy and the results were between 484.7 ± 413.4 and 1771.7 ± 776.1 CFU/m^3^ during different sampling times. In the present study, the seminar halls/lecture halls were sampled during the absence of students and results were within this range, indicating higher contamination of indoor air. These places were occupied occasionally only when the lecture had to be delivered to the large masses of students. The higher levels of bacteria even after the absences of students can be due to the cleaning frequency which was usually carried out before the lecture delivery during that particular day and the fact that the halls were always kept closed which limited the circulation of air from outside.

**Table 3:** Average bacteria indoor and outdoor counts at different sections of the university during the entire sampling period.

Sampling sites	Average bacteria counts (CFU/m^3^)						Average no. of occupants
	Total indoor bacteria	IGPB	IGNB	Total outdoor bacteria	OGPB	OGNB	
Classroom	1709	1123	488	1611	1180	529	0
Washroom	2647	1829	818	2613	1765	848	13
Canteen	2750	1911	839	2816	2015	801	35
Library	924	619	306	981	663	318	20
Environmental Lab	1998	1343	654	2028	1461	567	4
Seminar hall	1695	1145	550	1604	1120	485	0

*IGPB* indoor gram positive bacteria, *IGNB* indoor gram negative bacteria, *OGPB* outdoor gram positive bacteria, *OGNB* outdoor gram negative bacteria.

The average outdoor total bacteria count ranged between 981 and 2816 CFU/m^3^ at different sampling sites. Compared to all the sampling sites, canteen demonstrated higher average outdoor bacteria counts with levels of 2816 CFU/m^3^, succeeded by the washroom (2613 CFU/m^3^), environmental lab (2028 CFU/m^3^), classroom (1611 CFU/m^3^), seminar hall (1604 CFU/m^3^) and the lowest average outdoor bacteria counts were observed in the library (981 CFU/m^3^). For the canteen, the average bacteria level outside was higher than that inside. This is because the canteen was situated in close proximity to a road traffic, an outdoor playground, and a parking lot, all of which created continuous turbulence that allowed the dust particles to get suspended in the air. The average interior bacteria levels at the classroom, lavatory and seminar hall were greater than the outside average bacteria count, this could be because of continuous closure of doors and windows, which prevented airflow between the indoor and outdoor settings of the classroom and seminar hall and due to more availability of germs inside toilets and the wetness, which promotes the bacterial survival. Furthermore, the environmental lab and library's average outside bacteria levels were greater than the inside average bacteria count, it might be due the location of the environmental lab which was situated at the corner of the building, where there was insufficient airflow and the human movement outside the library as it was a common walk to reach to other floors and departments in the same building.

The method employed to cultivate the bacteria made it impossible to identify specific bacterial species, thus they were categorized as gram positive and gram negative. The dominant bacterial species identified during the study period both in the indoor and outdoor sampling locations are the gram positive bacteria and the levels of both gram positive bacteria and negative bacteria in inside and outside the sampling locations are presented in the Table 3. This finding is consistent with the studies of^8,45^ and may be attributed to have more resistant to external factors than the gram-negative bacteria, which is due to the presence of a thick, multilayered cell wall in gram-positive bacteria, which enables their survival, as reported in the studies of Refs.^20,46^. The low percentage of gram-negative bacteria detected can be due to bacterial damage during sampling. It is well known that some bacteria especially gram-negative bacteria can be damaged by the sampling technique^47^. Furthermore, the culture-based method does not account for viable but non-culturable microorganisms that still have the potential to cause disease^48^.

### Fungal bioaerosol concentration in indoor and outdoor air

In both indoor and outdoor air samples, fungus concentrations were lower than bacterial concentrations. Similar results were reported by^35^ examining air quality in the classroom, cafeteria and libraries in two schools of Poland. The average indoor and outdoor fungi counts are tabulated in the Table 4. The average indoor fungi count in different sections ranged between 656 and 1799 CFU/m^3^. In comparison with all the sections, canteen had the highest average indoor fungi counts of 1799 CFU/m^3^ proceeded by classroom (1388 CFU/m^3^), washroom (992 CFU/m^3^), environmental Lab (801 CFU/m^3^), seminar hall (728 CFU/m^3^) and the lowest average indoor fungi counts were found in the library (656 CFU/m^3^). Menteşe et al.^49^ studied bioaerosols levels in different indoor and outdoor environments in Ankara, Turkey and reported higher fungal concentrations in the kitchen. Current study showed a similar result indicating that the canteen had the peak average fungi count. The higher fungi levels can be due to high occupancy rates and it was noticed that the food preparation platform was open to the viewer and this can release the water vapor during cooking, thus resulting in favorable conditions for mold growth. Studies conducted by Ref.^50^ reported the average concentration of fungal aerosol in the indoor air being in the range of 78–788 CFU/m^3^. In Malaysia’s selected primary schools, the mean indoor fungal counts in the classroom without occupants were reported to be in the range of 65–530 CFU/m^3^. In Portuguese primary schools, a higher concentration ranges between 16 and 1792 CFU/m^3^ was reported by^51^. In our study, the average indoor fungi count from unoccupied classroom was a little higher than the Portuguese primary schools. Possible reasons for such high levels may be inadequate ventilation, as the classroom was kept completely closed and hygiene conditions. The fungi count in the toilets depends on many factors such as the type of the toilet, whether the switch toilet lid is open or closed and the flushing energy. The average bioaerosol concentration given by Ref.^52^ is approximately 300 CFU/m^3^. Kalwasińska et al.^27^ studies different sections in the library and the range of indoor fungi counts were between 13 and 933 CFU/m^3^. Present results for washroom were well within this range. An investigation of bioaerosol concentration during COVID-19 lock down period in a university of Turkey^53^, gave the values of the average indoor fungi count in environmental research center in the range of 96.06 ± 54.53 CFU/m^3^ and the study performed by Ref.^41^ reported values of 755 CFU/m^3^ for environmental lab. Our results were in agreement with the later study. Unoccupied seminar halls in the present study reported higher average indoor fungi count compared to the results given by Dang et al.^43^ which was ranging between 106.1 ± 81.3 CFU/m^3^ and 928.8 ± 398.1 CFU/m^3^ when the student occupancy was nearly 300. Inadequate cleaning schedule, enclosed indoor space and lack of circulation of air from the openings of the building might be some of the causes of higher average indoor counts in seminar halls^54^. Among the different sampling places, library showed lowest average indoor fungi counts. Similar results were reported by Refs.^35,49^ and the mean indoor fungi count of 543–876 CFU/m^3^ for main hall was reported by Ref.^27^ is in consistent with the current results.

**Table 4:** Average indoor and outdoor counts at different sampled locations.

Sampling sites	Average fungal concentrations CFU/m^3^	
	Indoor	Outdoor
Classroom	1388	1386
Washroom	992	1005
Canteen	1799	1619
Library	656	538
Environmental lab	801	805
Seminar hall	728	834

The average outdoor fungi count in different places ranged between 538 and 1619 CFU/m^3^. Among the different sampled locations, the canteen had the minimum average outdoor fungi counts of 1799 CFU/m^3^ then classroom (1388 CFU/m^3^), washroom (1005 CFU/m^3^), environmental Lab (805 CFU/m^3^), seminar hall (835 CFU/m^3^) and the lowest average indoor fungi counts were found in the library (834 CFU/m^3^). It is a general trend that the indoor fungal levels are always lower than the outdoor fungal counts due to lower relative humidity in the indoor environments, as reported by the literature. In the present study, out of six sampling locations, two sampling locations: canteen and library showed higher average fungi count and classroom had almost same average outdoor and indoor fungi counts. This difference in the two fungi air samples can be attributed to sampling time (1.30 pm to 2.30 pm), during which the occupancy levels inside the canteen and library will be at its peak. Rajasekar and Balasubramanian^23^ studied the bioaerosols levels in indoor and outdoor air of food courts and reported that the outdoor average fungi count during their peak hours was in the range 424 ± 10 CFU/m^3^. The study performed by Ref.^19^ in the outdoor air of bus station canteen during the afternoon hours, reported the total number of fungi of 8.2 × 10^2^ CFU/m^3^. Our study showed relatively higher values and this can be explained by the fact that this area is exposed to the influences of the nearby outdoor environments. In Poland, the bioaerosol in rural nursery schools were sampled and the average concentration of fungal aerosol in outdoor air of classroom was 623 CFU/m^3^ (range: 65–1792 CFU m^3^)^55^. The research carried out by Ref.^19^ for classroom at different levels showed a result of 680 CFU/m^3^ for the outside total number of fungus for the first floor classroom. The results of another research on outdoor fungal concentrations performed by Ref.^56^, ranged from 140 to 1055 CFU/m^3^. A higher mean outdoor fungal spore levels of 39–65,539 spores/m^3^ were observed by Ref.^57^. For the current study, the average outdoor fungi count in the outdoors of classroom reported a higher value of 1386 CFU/m^3^. This can be explained by the fact that outdoors of the classroom was open to the air and it was in close proximity to the open playground which influenced the outdoor fungal levels of classroom. Apart from the people, toilet bowl, washbasin and flushing of toilets will generate a great amount of microbes in the washrooms. The current investigation found that the average outdoor fungal count for washroom was greater than the inside values. This might be linked to a variety of factors, like the position of the washroom, which was in close proximity to the lift and surrounded by soil and plants; also, the fungus may have entered through the washroom door which was observed to be open during sampling. Umana et al.^58^ studied two different labs and the concentration of fungi was between 250 and 1790 CFU/m^3^ for indoor and outdoor air and the total number of fungi for labs studied by^19^ showed a values between 320 and 520 CFU/m^3^. The outcome of our study showed a higher value which is 805 CFU/m^3^ and it could be a result of its location being in the compacted area were there was little or no interference with the outdoor air. Our results for outdoor levels for lecture room was higher (834 CFU/m^3^) compared to the study conducted by^19^ (210–520 CFU/m^3^). Also the average outdoor fungi levels for seminar hall was more than the indoor levels. It could be due the sites placement which was near to the stairs and lift. Also, as the sampling period was in the afternoon (1.30 pm to 2.30 pm) the crowd near the lift might have contributed to higher outdoor levels. Some scholars^19,59,60^ have studied the outdoor fungi counts for library and the values given by respective scholars are 420–820 CFU/m^3^, 1530 CFU/m^3^ and 217–3750 CFU/m^3^ and out results are consistent with their findings.

In overall, many factors contribute to the bioaerosol emissions in the university campus. In the present study student density was the major factor which contributed to the bioaerosols emission in different environments. Higher bioaerosols levels were observed in places with high occupancy. Also, an interesting fact was that the seminar halls and the classrooms even being unoccupied showed higher levels of bioaerosols indoor and this might be due to the closure of doors and windows which restricted the entry of outdoor door air, the cleaning frequency and other miscellaneous factors.

### Predominant genera of airborne fungi

The different fungal species examined in the current study pertaining to both the indoor and outdoor air are *Cladosporium*,* Aspergillus niger*, *Pencillium*, *Rhizopus*, *Fusarium*, *Mucor*, *Alternaria*, *Nigrospora*,* Trichoderma*, *Aspergillus flavus, Neurospora and Aspergillus fumigates*. Among the different species, *Cladosporium* was found to be the most predominant fungi in both the environments. Spores of *Cladosporium* species are known to be widely dispersed in the air globally, and are the major airborne spores in temperate areas^35^. Our study results are consistent with other results^23,49,61–63^. Out of 772 taxa of *Cladosporium,* only 170 has recently been recognized as distinct species with *C. cladosporioides, C. herbarum, and C. sphaerospermum* being considered the three major species complexes^64^. Among them, *C. cladosporioides* has been found to cause Phaeohyphomycotic dermatitis in giant panda^65^. *Cladosporium* species have also been extensively described as opportunistic pathogenic organisms linked to several diseases in both humans and animals^66^. The presence of *Cladosporium* in the indoors of the studied locations might be due to their settlement in dust from outdoor air as they are primarily outdoor fungal taxa, commonly colonizing plant tissues. However, prior research revealed that CO_2_ levels and the number of inhabitants could have an impact on *Cladosporium* fungal presence in indoor environments^67^. It is possible that the aforementioned parameters, not measured in our work, played a role in sustaining the presence of *Cladosporium* in the studied sites through internal sources. *Aspergillus niger* was the second most abundant fungal species in the indoor and outdoor air. *A. niger*, which can grow on a wide range of materials, is a frequent contaminant of food, soil, and indoor environments. Although its spores are widespread, the fungus has been reported to a less likely cause of human disease in comparison to other *Aspergillus species*^68^. Normal invasion of tissues by *Aspergillus niger* occurs when those tissues have previously been made vulnerable by bacterial infections, physical trauma, or a buildup of cerumen in the external auditory canal. Otomycosis, a superficial fungal infection in the ear, throat, or nose that can be subacute or chronic, is caused by Aspergillus species, including *A. niger*^69^. The *A. niger* in indoors could be due to their growth on polluted ventilation systems and on wet construction materials. The third most abundant fungi in both the environments was *Pencillium. Penicillium species* are numerous and extensively spread in the environment, yet despite their number and variety, they are not frequently linked to illnesses in people and animals^70^. *Penicillium species* are numerous and widely distributed in the dust of indoor environment, but despite this, they are not frequently linked to human and animal infections. The presence of *Penicillium* in the interiors of the sampled locations could be from transport of outdoor air, their growth on the building material to obtain nutrients for growth and the presence of sufficient moisture and low relative humidity which favors their survival indoors. In the present study they were relatively higher in indoors than outdoors. Of the few pathogenic species that affect plants, *P. citrinum, P. chrysogenum, P. digitatum, P. expansum, and P. marneffei* are frequently linked to both humans and animals, with inhalation and occasionally ingestion being the main routes of infection^71,72^. Penicilliosis is the term used to describe illnesses that are caused by any *Penicillium species*. Infections including keratitis, endophtalmitis, otomycosis, pneumonia, endocarditis, and urinary tract infections have all been linked to species in this genus^73^. *Rhizopus* is *extensively* present on grains, fruits, and vegetables that have been preserved, in the air or in compost, as well as in a variety *of* restricted indoor conditions. Numerous investigations of the air spore in libraries have shown that *Rhizopus* species grow on paper^74^. *Rhizopus* *species* have a high moisture requirement for growth and are known to cause diseases systemic diseases^59^. *Fusarium* is commonly found occurring in the soil and in association with plants^75^. The common *Fusarium* infections are keratitis and onychomycosis with other less common conditions such as sinusitis, pneumonia, thrombophlebitis and fungemia and infection can occur through inhalation of air-borne conidia and through cuts/breaks in the skin^76^. The other species were in negligible amounts when compared to the five most abundant species. Among the several Alternaria species, *A. alternata* is renowned as a ubiquitous and prevalent allergic fungus that causes IgE-mediated respiratory illnesses, notably asthma exacerbations^77^. Indoor air fungus development is primarily influenced by moisture and the presence of carbon sources. Controlling moisture levels and lowering indoor organic pollutants are thus the two most crucial methods for limiting or eliminating fungal development^78^. Indoor fungal count is decreased via mechanical ventilation, forced air heating, dehumidifiers, air filters, and air conditioners^79^. The control of excessive moisture and consequent fungal development depends on the design, construction, and upkeep of building envelopes^80^.

### Indoor-to-outdoor ratio of bioaerosol concentration

There is no consensus on how to define a specific bioaerosol limit value for interior spaces, despite the presence of several established indoor air quality guidelines for bioaerosol levels. The lowest amount that might harm human health has not yet been precisely determined. When bioaerosol sampling is conducted in an indoor setting, an outdoor bioaerosol sample should be acquired to compare the influence of the inside and outside environments. The source location of the bioaerosol is determined using the indoor-to-outdoor ratio (I/O). If I/O > 1, the source is from the indoor environment, and the outdoor bioaerosol source differs from the indoor bioaerosol source^49^. Table 5 displays the I/O ratios for bacterial and fungal counts that were determined for each site. The I/O ratios for airborne bacteria in the toilet and seminar room were > 1, indicating that several factors, including human presence, poor sanitation, toilet flushing, and incorrect air circulation system maintenance, contribute to the high indoor bacterial count. The classroom, canteen, library, and environmental laboratory showed an I/O ratio of < 1, indicating the lack of bacterial contamination from the outdoor environment. The I/O ratio for fungi was > 1 in the classroom, washroom, canteen, library, and environmental lab, indicating that they are the major sources of fungi are likely internal, such as building occupants, as well as the building materials that host the fungi growth. In the classroom fungi might have been retained on the wooden benches. Dampness near the washbasins of the washroom could be one of the internal fungal source, as it is reported by many researchers that the survival of most fungi require moisture content. At canteen the occupants could be the source for indoor fungi counts, as they were at the maximum occupancy loads during the sampling period. The problem source in the indoor library are the cellulose material like paper on which the molds are likely to proliferate. Hence, people exposed to these environments are at a high risk of developing fungal diseases.

**Table 5:** Indoor-to-outdoor ratio of bioaerosol concentration.

Location	Bacteria	Fungi
Classroom	0.94	1.00
Washroom	1.01	1.03
Canteen	0.98	1.11
Library	0.94	1.22
Environmental lab	0.99	1.00
Seminar hall	1.06	0.87

**Figure 3:**
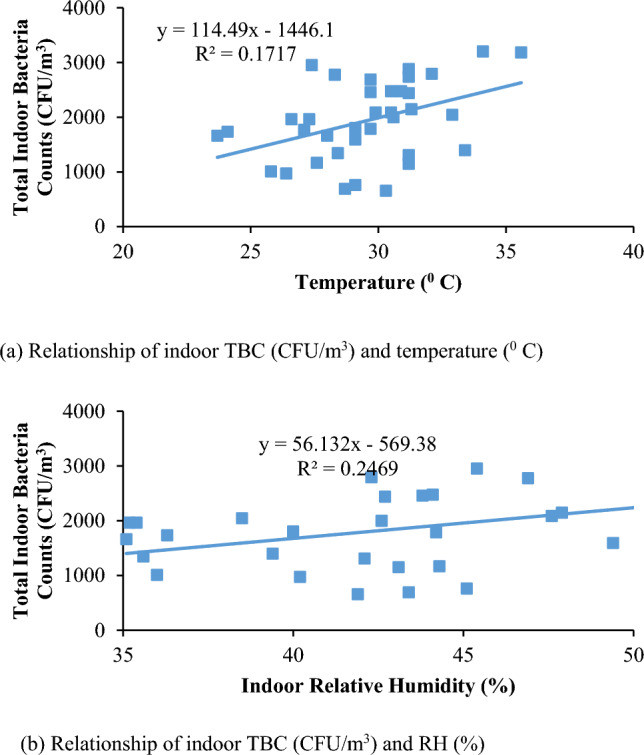
Plot showing the relationship between indoor TBC (CFU/m^3^) and environmental conditions.

**Figure 4:**
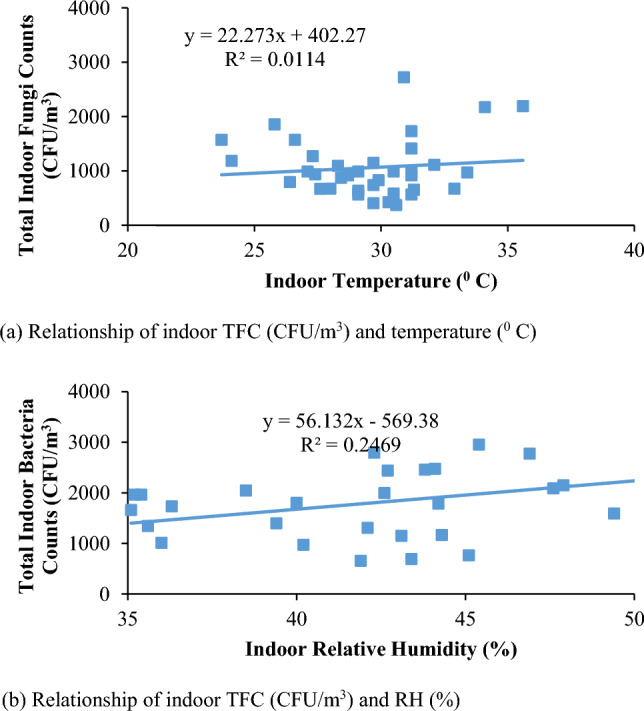
Plot of indoor TFC (CFU/m^3^) and environmental conditions.

Correlation between indoor bioaerosol concentration, and indoor temperature and indoor relative humidity were not significant. As shown in Fig. 3, there is no significant influence of indoor temperature and relative humidity on indoor TBC (R^2^ = 0.1717 in Fig. 3a, 0.2469, Fig. 3b, respectively for temperature and relative humidity). Similarly, as shown in Fig. 4,it can be seen that no significant correlation exists for indoor temperature and relative humidity over indoor Total Fungal Count (TFC) where R^2^ values are 0.0114 (Fig. 4a), and 0.2469 (Fig. 4b), respectively.

Therefore, the analysis results show that the temperature and relative humidity has an insignificant impact on overall indoor bioaerosol in the University indoor environment, but could be because of various indoor sources.

## Conclusion

The bacterial and fungal aerosol concentrations at all locations exceeded the stipulated guideline limit by the American Industrial Hygiene Association. The canteen showed the highest concentration of viable bacteria and fungi both indoors and outdoors due to the presence of more occupants, favorable meteorological factors, and poor hygiene. The second highest concentration of bacterial and fungal aerosols was observed in the washrooms, as frequent flushing and dampness enable the survival of bioaerosols. The low bacteria and fungi counts in both the environments of the library can be attributed to the fact that the it had a vast area and the sampling was done in the center of the room, good hygienic conditions and proper maintenance of the ventilation systems Gram-positive bacteria were more predominant in both indoor and outdoor environments compared to gram-negative bacteria. The fungal species isolated and identified from indoor and outdoor sites were *Cladosporium*, *Aspergillus niger*, *Penicillium*, *Rhizopus*, *Fusarium*, *Mucor*, *Alternaria*, *Nigrospora*, *Trichoderma*, *Neurospora*, *Aspergillus fumigatus*, and *Aspergillus flavus*. Among these, the most predominant was *Cladosporium*, followed by *Aspergillus niger*, *Penicillium*, *Rhizopus*, *Fusarium*, and *Mucor*. The other genera were present at negligible concentrations. Isolated fungi were linked to respiratory tract illnesses and allergies. Identification and elimination of the microbial source, equipment maintenance, humidity control, natural ventilation, use of filters in ventilation, good sanitary practices, and air cleaning using disinfectants and biocides are some of the mitigation steps that should be implemented to prevent the negative health effects of bioaerosols.

## Data Availability

All the data collected or analyzed during the current investigation are included in this published article.

## References

[1] Ingole SP, Deshmukh V (2020). Bioaerosols load in indoor and outdoor environment: A brief review. J. Appl. Res..

[2] Lindemannt J, Upper CD (1985). Aerial dispersal of epiphytic bacteria over bean plants. Appl. Environ. Microbiol..

[3] Cullinan P, Cook A, Nieuwenhuijsen MJ, Sandiford C, Tee RD, Venables KM, McDonald JC, Newman Taylor AJ (2001). Allergen and dust exposure as determinants of work-related symptoms and sensitization in a cohort of flour-exposed workers; A case-control analysis. Ann. Occup. Hyg..

[4] Kumari S, Gond DK, Samuel CO, Abassi P (2011). A comparative study of aeromycospora in different localities of Gorakhpur. UP. Indian J. Sci. Res..

[5] Karwowska E (2005). Microbiological air contamination in farming environment. Pol. J. Environ. Stud..

[6] Kumar, P., Kausar, M. A., Singh, A. B., & Singh, R. Biological contaminants in the indoor air environment and their impacts on human health. In *Air Quality, Atmosphere and Health* (Vol. 14, Issue 11, pp. 1723–1736). Springer Science and Business Media B.V. 10.1007/s11869-021-00978-z (2021). 10.1007/s11869-021-00978-z PMC8346343 34394766

[7] Jaenicke R (2005). Abundance of cellular material and proteins in the atmosphere.

[8] Mandal J, Brandl H (2011). Open access bioaerosols in indoor environment—a review with special reference to residential and occupational locations. Open Environ. Biol. Monit. J..

[9] Ferguson RMW, Neath CEE, Nasir ZA, Garcia-Alcega S, Tyrrel S, Coulon F, Dumbrell AJ, Colbeck I, Whitby C (2021). Size fractionation of bioaerosol emissions from green-waste composting. Environ. Int..

[10] Lee JH, Jo WK (2006). Characteristics of indoor and outdoor bioaerosols at Korean high-rise apartment buildings. Environ. Res..

[11] Huang HL, Lee MK, Shih HW (2017). Assessment of indoor Bioaerosols in public spaces by real-time measured airborne particles. Aerosol Air Qual. Res..

[12] Foarde, K., Dulaney, P., Cole, E., VanOsdell, D., & Ensor, D. Assessment of fungal growth on ceiling tiles under environmentally characterized conditions (Issue 22) (1993).

[13] Pasanen AL, Kasanen JP, Rautiala S, Ikäheimo M, Rantamäki J, Kääriäinen H, Kalliokoski P (2000). Fungal growth and survival in building materials under fluctuating moisture and temperature conditions. Int. Biodeter. Biodegr..

[14] Kulmala M, Asmi A, Pirjola L (1999). Indoor air aerosol model: The effect of outdoor air, filtration and ventilation on indoor concentrations. Atmos. Environ..

[15] Hayleeyesus SF, Ejeso A, Derseh FA (2015). Quantitative assessment of bio-aerosols contamination in indoor air of University dormitory rooms. Int. J. Health Sci..

[16] Rintala H, Pitkäranta M, Toivola M, Paulin L, Nevalainen A (2008). Diversity and seasonal dynamics of bacterial community in indoor environment. BMC Microbiol..

[17] Sidra S, Ali Z, Sultan S, Ahmed S, Colbeck I, Nasir ZA (2015). Assessment of airborne microflora in the indoor micro-environments of residential houses of lahore, pakistan. Aerosol Air Qual. Res..

[18] Mouli PC, Mohan SV, Reddy SJ (2005). Assessment of microbial (bacteria) concentrations of ambient air at semi-arid urban region: Influence of meteorological factors. Appl. Ecol. Environ. Res..

[19] Bomala, K., & Saramanda, G. *Microbiological indoor and outdoor air quality Visakhapatnam city, India*. http://www.journalcra.com (2016).

[20] Chan DWT, Leung PHM, Tam CSY, Jones AP (2008). Survey of airborne bacterial genus at a university campus. Indoor Built Environ..

[21] Er, C. M., Sunar, N. M., Leman, A. M., Khalid, A., Ali, R., Zaidi, E., & Azhar, A. T. S. Indoor and outdoor surface-growing fungi contamination at higher institutional buildings in a Malaysian University. *IOP Conf. Ser. Earth Environ. Sci.* **140**(1):1. 10.1088/1755-1315/140/1/012118 (2018).

[22] Balasubramanian R (2012). Airborne bacteria, fungi, and endotoxin levels in residential microenvironments: A case study. Aerobiologia.

[23] Rajasekar A, Balasubramanian R (2011). Assessment of airborne bacteria and fungi in food courts. Build. Environ..

[24] Heath GA (2005). Do indoor pollutants and thermal conditions in schools influence student performance? A critical review of the literature. Indoor Air.

[25] Daisey, J. M. Indoor air quality, ventilation and health symptoms in schools: An analysis of. *Indoor Air LBNL-48287*. https://www.osti.gov/servlets/purl/828725 (2003). 10.1034/j.1600-0668.2003.00153.x 12608926

[26] Hayleeyesus, S. F., & Manaye, A. M. Microbiological quality of indoor air in University libraries. *Asian Pacific J. Trop. Biomed.* **4**(Suppl 1), S312–S317. 10.12980/APJTB.4.2014C807 (2014). 10.12980/APJTB.4.2014C807 PMC4025286 25183103

[27] Kalwasińska A, Burkowska A, Wilk I (2012). Microbial air contamination in indoor environment of a University Library. Ann. Agric. Environ. Med..

[28] Bhangar S, Huffman JA, Nazaroff WW (2014). Size-resolved fluorescent biological aerosol particle concentrations and occupant emissions in a university classroom. Indoor Air.

[29] Lia Chatzidiakou, D. M. & Alex J. S. What do we know about indoor air quality in school classrooms? A critical review of the literature (2012).

[30] Grisoli P, Rodolfi M, Chiara T (2012). Evaluation of microbiological air quality and of microclimate in university classrooms. Environ. Monit. Assess..

[31] Qian J, Hospodsky D, Yamamoto N, Nazaroff WW, Peccia J (2012). Size-resolved emission rates of airborne bacteria and fungi in an occupied classroom. Indoor Air.

[32] Nunes I, Mesquita N, Cabo Verde S, Bandeira AML, Carolino MM, Portugal A, Botelho ML (2013). Characterization of an airborne microbial community: A case study in the archive of the University of Coimbra, Portugal. Int. Biodeterior. Biodegr..

[33] Di Giulio M, Grande R, Di Campli E, Di Bartolomeo S, Cellini L (2010). Indoor air quality in university environments. Environ. Monit. Assess..

[34] Priyamvada H, Priyanka C, Singh RK, Akila M, Ravikrishna R, Gunthe SS (2018). Assessment of PM and bioaerosols at diverse indoor environments in a southern tropical Indian region. Build. Environ..

[35] Małecka-Adamowicz M, Koim-Puchowska B, Dembowska EA (2020). Diversity of bioaerosols in selected rooms of two schools and antibiotic resistance of isolated staphylococcal strains (Bydgoszcz, poland): A case study. Atmosphere.

[36] Jeong SB, Ko HS, Heo KJ, Shin JH, Jung JH (2022). Size distribution and concentration of indoor culturable bacterial and fungal bioaerosols. Atmos. Environ..

[37] Li Y, Wang W, Guo X, Wang T, Fu H, Zhao Y, Wang W (2015). Assessment of airborne bacteria and fungi in various university indoor environments: A case study in Chang’an University China. Environ. Eng. Sci..

[38] Lou M, Liu S, Gu C, Hu H, Tang Z, Zhang Y, Xu C, Li F (2021). The bioaerosols emitted from toilet and wastewater treatment plant: A literature review. Environ. Sci. Pollut. Res..

[39] Chan WY, Mui MKW, Wong LT (2009). Airborne bacteria assessment in an office washroom. Conf. Green Build. Towards Eco-City.

[40] Hospodsky D, Qian J, Nazaroff WW, Yamamoto N, Bibby K, Rismani-Yazdi H, Peccia J (2012). Human occupancy as a source of indoor airborne bacteria. PLoS ONE.

[41] Srivastava A, Singh M, Jain VK (2012). Identification and characterization of size-segregated bioaerosols at Jawaharlal Nehru University New Delhi. Nat. Hazards.

[42] Bartlett KH, Kennedy SM, Brauer M, Van Netten C, Dill B (2004). Evaluation and determinants of airborne bacterial concentrations in school classrooms. J. Occup. Environ. Hyg..

[43] Dang, D. Y. N., Vuong, H. N., Nguyen, T. T. Microbiological contamination of indoor air in university classrooms(Case study: University of Science - Vietnam National University,Ho Chi Minh city). *Vietnam J. Sci. Technol. Eng.* **62**(4), 30–35. 10.31276/vjste.62(4).30-35 (2020).

[44] Mainka, A., Zajusz-Zubek, E., Kozielska, B., & Bragoszewska, E. Investigation of air pollutants in rural nursery school - A case study. *E3S Web Conf.* **28**, 1–8. 10.1051/e3sconf/20182801022 (2018).

[45] Kumari H, Chakraborti T, Singh M, Chakrawarti MK, Mukhopadhyay K (2020). Prevalence and antibiogram of coagulase negative Staphylococci in bioaerosols from different indoors of a university in India. BMC Microbiol..

[46] Abosede Sarah A (2017). Indoor and outdoor concentrations of bioaerosols and meteorological conditions of selected salons in four areas of Ibadan North Local Government Area. Int. J. Environ. Monit. Ana..

[47] Chang CW, Chou FC (2011). Methodologies for quantifying culturable, viable, and total Legionella pneumophila in indoor air. Indoor Air.

[48] D’Arcy N, Canales M, Spratt DA, Lai KM (2012). Healthy schools: Standardisation of culturing methods for seeking airborne pathogens in bioaerosols emitted from human sources. Aerobiologia.

[49] Menteşe S, Arisoy M, Rad AY, Güllü G (2009). Bacteria and fungi levels in various indoor and outdoor environments in Ankara, Turkey. Clean Soil Air Water.

[50] Mainka A, Brągoszewska E, Kozielska B, Pastuszka JS, Zajusz-Zubek E (2015). Indoor air quality in urban nursery schools in Gliwice, Poland: Analysis of the case study. Atmos. Pollut. Res..

[51] Madureira J, Paciência I, De Oliveira Fernandes E (2012). Levels and indoor-outdoor relationships of size-specific particulate matter in naturally ventilated portuguese schools. J. Toxicol. Environ. Health Part A Curr. Issues.

[52] Knowlton SD, Boles CL, Perencevich EN, Diekema DJ, Nonnenmann MW (2018). Bioaerosol concentrations generated from toilet flushing in a hospital-based patient care setting. Antimicrob. Resist. Infect. Control.

[53] Bhat MA, Eraslan FN, Awad A, Malkoç S, Üzmez ÖÖ, Döğeroğlu T, Gaga EO (2022). Investigation of indoor and outdoor air quality in a university campus during COVID-19 lock down period. Build. Environ..

[54] Ding W, Li L, Han Y, Liu J, Liu J (2016). Site-related and seasonal variation of bioaerosol emission in an indoor wastewater treatment station: Level, characteristics of particle size, and microbial structure. Aerobiologia.

[55] Bragoszewska E, Mainka A, Pastuszka JS (2016). Bacterial and fungal aerosols in rural nursery schools in Southern Poland. Atmosphere.

[56] Hussin NHM, Sann LM, Shamsudin MN, Hashim Z (2011). Characterization of bacteria and fungi bioaerosol in the indoor air of selected primary schools in Malaysia. Indoor Built Environ..

[57] Levetin E, Shaughnessy R, Fisher E, Ligman B, Harrison J, Brennan T (1995). Indoor air quality in schools: Exposure to fungal allergens. Aerobiologia.

[58] Umana S, Uko MP, Agbo BE, Bassey M, Edet N, Uko M, Agbo B (2018). Microbiological quality of indoor and outdoor air within biological sciences laboratories in Akwa Ibom State University. Nigeria. Front. Environ. Microbiol..

[59] Ghosh B, Lal H, Kushwaha R, Hazarika N, Srivastava A, Jain VK (2013). Estimation of bioaerosol in indoor environment in the university library of Delhi. Sustain. Environ. Res..

[60] Uzoechi AU, Obi TNN, Nnagbo PC, Ohalete CN, Anyiam VI (2017). Microbiological evaluation of indoor air quality of state university library. Asian J. Appl. Sci..

[61] Lu Y, Wang X, Almeida LCS, d. S., & Pecoraro, L. (2022). Environmental factors affecting diversity, structure, and temporal variation of airborne fungal communities in a research and teaching building of Tianjin University China. J. Fungi.

[62] Nageen Y, Asemoloye MD, Põlme S, Wang X, Xu S, Ramteke PW, Pecoraro L (2021). Analysis of culturable airborne fungi in outdoor environments in Tianjin China. BMC Microbiol..

[63] Yuan C, Wang X, Pecoraro L (2022). Environmental factors shaping the diversity and spatial-temporal distribution of indoor and outdoor culturable airborne fungal communities in Tianjin University Campus, Tianjin China. Front. Microbiol..

[64] Bensch K, Groenewald JZ, Meijer M, Dijksterhuis J, Jurjevi Z, Andersen B, Houbraken J, Crous PW, Samson RA (2018). Cladosporium species in indoor environments. Stud. Mycol..

[65] Ma X, Gu Y, Liu X, Li D, Ling S, Hou J, Wang C, Cao S, Huang X, Wen X, Ruan J, Dong C, Li C, Tong Y (2013). Phaeohyphomycotic dermatitis in a giant panda (Ailuropoda melanoleuca) caused by Cladosporium cladosporioides. Med. Mycol. Case Rep..

[66] Sandoval-Denis M, Sutton DA, Martin-Vicente A, Cano-Lira JF, Wiederhold N, Guarro J, Gené J (2015). Cladosporium species recovered from clinical samples in the United States. J. Clin. Microbiol..

[67] Heudorf U, Neitzert V, Spark J (2009). Particulate matter and carbon dioxide in classrooms—The impact of cleaning and ventilation. Int. J. Hyg. Environ. Health.

[68] Mousavi B, Hedayati MT, Hedayati N, Ilkit M, Syedmousavi S (2016). Aspergillus species in indoor environments and their possible occupational and public health hazards. Curr. Med. Mycol..

[69] Person AK, Chudgar SM, Norton BL, Tong BC, Stout JE (2010). Aspergillus niger: An unusual cause of invasive pulmonary aspergillosis. J. Med. Microbiol..

[70] Mok T, Koehler AP, Yu MY, Ellis DH, Johnson PJ, Wickham NWR (1997). Fatal Penicillium citrinum pneumonia with pericarditis in a patient with acute leukemia. J. Clin. Microbiol..

[71] Imwidthaya P, Thipsuvan K, Chaiprasert A, Danchaivijitra S, Sutthent R, Jearanaisilavong J (2001). Penicillium marneffei: Types and drug susceptibility. Mycopathologia..

[72] Walsh TJ, Groll A, Hiemenz J, Fleming R, Roilides E, Anaissie E (2004). Infections due to emerging and uncommon medically important fungal pathogens. Clin. Microbiol. Infect..

[73] Deshpande SD (1999). A study of mycotic keratitis in Mumbai. Indian J. Pathol. Microbiol..

[74] Richardson MD, Rautemaa-Richardson R (2020). Biotic environments supporting the persistence of clinically relevant mucormycetes. J. Fungi.

[75] Georgiadou SP, Velegraki A, Arabatzis M, Neonakis I, Chatzipanagiotou S, Dalekos GN, Petinaki E (2014). Cluster of Fusarium verticillioides bloodstream infections among immunocompetent patients in an internal medicine department after reconstruction works in Larissa, Central Greece. J. Hosp. Infect..

[76] Nucci M, Anaissie E (2007). Fusarium infections in immunocompromised patients. Clin. Microbiol. Rev..

[77] Downs SH, Mitakakis TZ, Marks GB, Car NG, Belousova EG, Leüppi JD, Xuan W, Downie SR, Tobias A, Peat JK (2001). Clinical importance of Alternaria exposure in children. Am. J. Respir. Crit. Care Med..

[78] Oppliger A, Charrière N, Droz PO, Rinsoz T (2008). Exposure to bioaerosols in poultry houses at different stages of fattening; use of real-time PCR for airborne bacterial quantification. Ann. Occup. Hygiene.

[79] Busse PJ, Farzan S, Cunningham-rundles C (2007). Health effects of indoor fungi. Immunology.

[80] Soldatova LN, Rocca-Serra P, Dumontier M, Shah NH (2014). WHO guidelines for indoor air quality: Dampness and mould. J. Biomed. Seman..

